# PRG: A Distance Measurement Algorithm Based on Phase Regeneration

**DOI:** 10.3390/s18082595

**Published:** 2018-08-08

**Authors:** Yansong Cui, Di Zhu, Yanxu Liu

**Affiliations:** School of Electronic Engineering, Beijing University of Posts and Telecommunications, No. 10 Xitucheng Road, Beijing 100876, China; cuiys@bupt.edu.cn (Y.C.); cordialz@bupt.edu.cn (D.Z.)

**Keywords:** IoT, phase regeneration, distance measurement, GBDT

## Abstract

With the booming development of the Internet of things (IoT) industry, the demand of positioning technology in various IoT application scenarios is also greatly increased. To meet the positioning requirements of the IoT application, we propose a distance measurement method based on phase regeneration that can provide positioning capability for IoT applications in indoor and outdoor environments. The PRG algorithm consists of two phases: coarse ranging phase and fine ranging phase. Fingerprint positioning algorithm based on Gradient Boost Decision Tree (GBDT) is used to determine coarse distance. The host machine measures the difference between the transmitted carrier phase and the received regenerative carrier phase to fix the fine distance and then the coarse distance is used to determine the carrier phase integer ambiguity. Finally, high precision ranging is realized. Simulation results show that the PRG method can achieve range finding with decimeter level precision under the 10 MHz subcarrier frequency.

## 1. Introduction

Positioning of node information is crucial in IoT applications. In plenty of IoT applications, the sensor node information is meaningless when the positioning is uncertain, such as wearable devices, sweeping robot et.al. Location-aware based on range finding can be widely used in IoT node [1,2], compared with based on range-free has higher positioning accuracy [3]. Therefore, it is very important to nodes positioning to study the distance measurement of IoT nodes in the IoT.

Distance measurement is mainly divided by two methods: based on received signal strength (RSS) and time-of-flight estimation [4]. Wang H. et al. proposed a novel range finding algorithm based on RSSI in wireless sensor network ,the algorithm by creating a database between RSSI readings and distance reduce the impact on precision of range finding due to RSSI measurements volatility [5]. Cho H. et al. proposed a distance measure method by bluetooth equipment in the IoT, and the method by adding extra self-calibration beacon reduce the influence of environmental variation on the precision of range finding , and 90% probability of accuracy of range finding is less than 1.5 m [6]. Chowdhury T. I. et al. have used a multi-step RSSI-based distance calculation model using Samsung Galaxy S4 smartphone. They achieved the error level up to 13.4% [7]. Barai S. et al. proposed a method of distance measurement based on the curve fitting between RSSI and distance, reduce the average error level up to 8.32% [8]. Akiyama T. et al. proposed a positioning method of using smart phone to measure the time of arrival(TOA), employ modulated light with an signal for the time-of-flight short measurement, CMOS sensor to achieve precise TOA measurements by mobile phones, can realize positioning error less than 10 mm [9]. However, a low cost and simple distance measurement technology is still needed to provide the accuracy of meter positioning [10].

To address the aforementioned shortcomings, we present phase regeneration method for distance measurement, combining coarse range and fine range of ways to meet the demand of the system is simple, high precision range finding. Different positioning requirements can choose different parameters making the method is suitable for many positioning scenarios. Therefore, the range finding method based on phase regeneration has greater flexibility and scalability.

The remainder of this paper is organized as follows: A detailed description of PRG method for distance measure is presented in Section 2. In Section 3, the simulation results and evaluation is listed. Section 4 concludes this paper.

## 2. Principle of PRG

### 2.1. Phase Regeneration Ranging Algorithm

**Figure 1:**
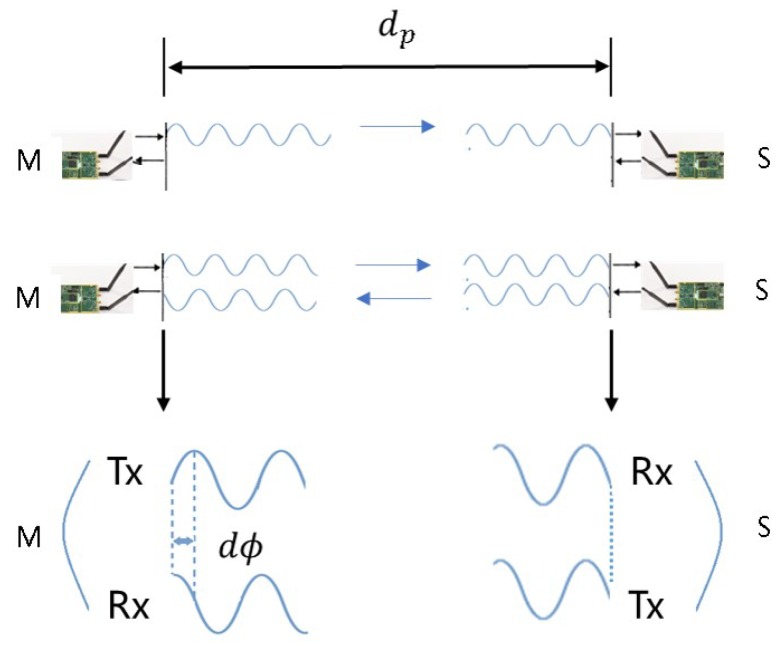
PRG range finding model.

The PRG algorithm range finding model is shown in Figure 1. The detailed process of the PRG algorithm is as follows: Firstly, the pseudo-range measurement $d_{p}$ between the master node M and the slave node S can be obtained from the coarse range finding method. Secondly, the master node M continuously transmits the range finding signal to slave node S. After the slave node S receives the range finding signal and synchronizes the phase and frequency through the carrier tracking loop, then the slave node S continuously transmits the replication range finding signal to master node M. Thirdly, the master node M receives its replication range finding signal from previous slave node S and then obtains the phase difference measurement $d_{\phi }$ between the transmitted and the received replication carrier. Finally, the distance *d* between the master and slave node can be expressed by:

(1) $$d=\lambda \cdot \left(\frac{1}{2}d\phi +{\left\lfloor \frac{d_{p}}{2\lambda }\right\rfloor }_{floor}\right)$$

where λ is the wavelength of range finding signal, ${\left\lfloor \cdot \right\rfloor }_{floor}$ represents round down function. In the ideal case, it can be seen from the above Equation (1) that high precision range finding can be achieved without coarse range finding phase when the distance between master and slave node is less than $\frac{\lambda }{2}$. In addition, the PRG method is unavailable due to carrier phase integer ambiguity in Equation (1) when the error of coarse range finding is greater than $\frac{\lambda }{2}$. A conservative estimation method for carrier-tracking loop error is to ensure that the 1σ tracking error is no more than $\frac{\lambda }{24}$. We assume that carrier-tracking loop error of the master and slave node is independent and the condition of no carrier phase integer ambiguity is that the precision of coarse range finding is $d_{p}\leq \frac{5\lambda }{12}$.

### 2.2. Range Finding Signal

**Figure 2:**
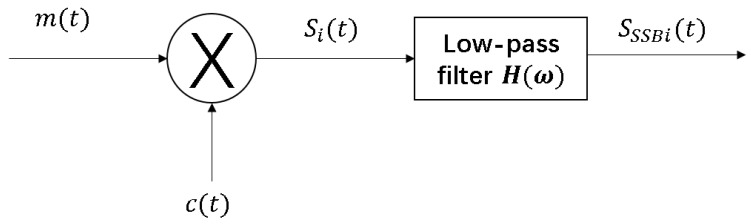
Range finding signal modulation block diagram.

The range finding signal of PRG algorithm is single-sideband modulation. The modulation block diagram is shown in Figure 2, first the double sideband signal is obtained from the analog baseband signal m(t) with mean 0 and sinusoidal carrier signal c(t) multiplication and then the amplitude modulated signal by low pass filter H(ω) for filter, get lower sideband modulation signal $S_{SSBi}\left(t\right)$. The analog baseband signal in PRG algorithm is tone signal with a frequency of $f_{d}$, and denoted by:

$$m\left(t\right)=2A\cdot \sin2\pi f_{d}$$

where 2A represents the amplitude of analog baseband signal. We can get the signal $S_{i}\left(t\right)$ as:

$$S_{i}\left(t\right)=2A\cdot \sin2\pi f_{d}t\cdot \sin2\pi f_{i}t=A\cdot \left[\cos2\pi (f_{i}-f_{d})t-\cos2\pi (f_{i}+f_{d})t\right]$$

The range finding signal $s_{SSBi}\left(t\right)$ is obtained by a low-pass filtering of $S_{i}\left(t\right)$ with cutoff frequency of $f_{i}$ and can be expressed as:

$$s_{SSBi}\left(t\right)=A\cdot \cos2\pi \left(f_{i}-f_{d}\right)t$$

The modulation signal can be got with frequency of $f_{1}-f_{d},f_{2}-f_{d},\cdots ,f_{n}-f_{d}$ and different carrier frequencies are used to identify different master and slave node to apply multi-master, multi-slave case. To ensure that each modulation signal does not interfere with each other, we need to make sure that adjacent two frequency points have a wide enough frequency protection interval so that the RF front end can separate signals from each frequency point.

### 2.3. Range Finding Signal Acquisition

#### 2.3.1. Search Scope

The doppler shift can be ignored because the current user case of the scenario is low dynamic. Therefore, the search space of range finding signal is one dimension, the signal acquisition can be carried out by searching the frequency point of the signal.

#### 2.3.2. Signal Detection

**Figure 3:**
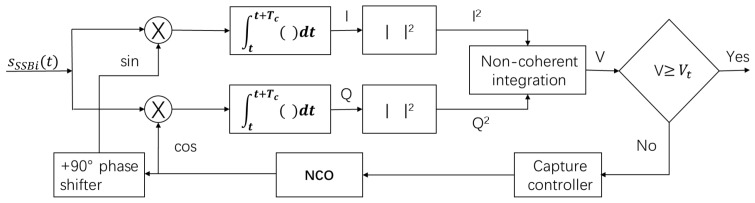
Typical of signal acquisition block diagram.

Signal acquisition is estimate the frequency of the receiving signal and then initialize the tracking loop according to these parameter to help the receiving channel to track the signal. Figure 3 illustrates a high level block diagram typical of signal acquisition where the digitized received RF signal is applied to the input. Referring to Figure 3, first the digital RF is stripped of the carrier by the replica carrier signals to produce in-phase (i) and quadraphase (q) sampled data and then the results i and q generate data to I and Q after the coherent integration with time $T_{c}$. Finally, the incoherent integral amplitude *V* is obtained by incoherent integration. The incoherent detection method of signal acquisition determines whether the received signal has been detected by detecting the magnitude of the incoherent integral amplitude *V*: if the incoherent integral amplitude *V* is less than the acquisition threshold value $V_{t}$, the signal has not been searched and the receiver adjusts the carrier numerical control oscillator according to the set search step and continues to search and detect signals in the next search cell. Otherwise, if $V>V_{t}$, the signal is searched, and the receiver then confirms that the signal is successfully captured.

The complex form of coherent integration is:

$$r\left(n\right)=I\left(n\right)+jQ\left(n\right)=a\cdot \mathrm{sinc}\left(f_{e}T_{c}\right)\cos\phi_{e}+n_{I}+j\left[a\cdot \mathrm{sinc}\left(f_{e}T_{c}\right)\sin\phi_{e}+n_{Q}\right]$$

Where *a* is the amplitude of signal, $f_{e}$ represents frequency difference between received carrier and replica carrier generated by NCO, $\phi_{e}$ represents phase difference between 2 carriers, and $n_{I}∼N\left(0,\sigma_{n}^{2}\right)$, $n_{Q}∼N\left(0,\sigma_{n}^{2}\right)$, and $n_{Q}$ and $n_{I}$ is independent. The $\sigma_{n}$ can be expressed by:

$$\sigma_{n}=\frac{N_{0}}{T_{c}}$$

Without noise, the amplitude of r(n) is:

$$V=\left|r\left(n\right)\right|=\sqrt{I^{2}\left(n\right)+Q^{2}\left(n\right)}=a\cdot \left|sinc\left(f_{e}T_{c}\right)\right|$$

**Figure 4:**
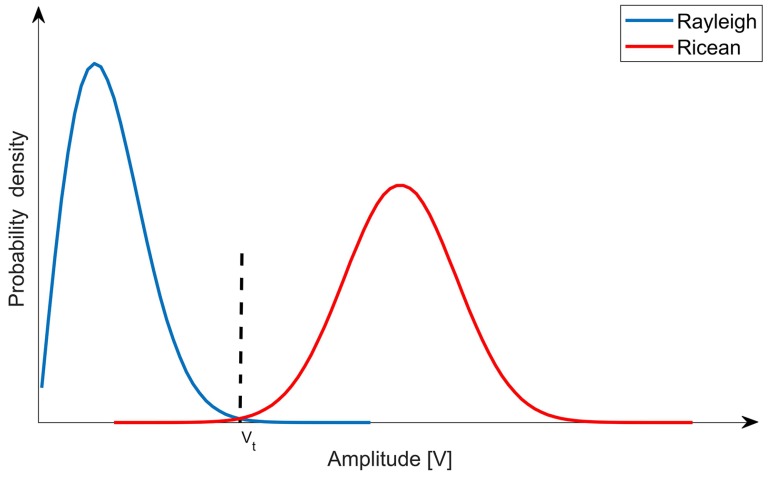
Incoherent integral probability density.

We can see phase difference $\phi_{e}$ between receiving and replica carrier does not affect the value of *V*, so the *V* can be used for capture detection. From the above assumption of $n_{I}$ and $n_{Q}$, the amplitude *V* have Ricean distributions in the presence signal and have Rayleigh distributions in absence of signals [11]. The Rayleigh distribution probability density function $f_{n}\left(v\right)$ is shown in Equation (2) and the probability density function $f_{s}\left(v\right)$ of Ricean distribution is shown in Equation (3), and its probability density curve is shown in Figure 4.

(2) $$f_{n}\left(v\right)=\frac{v}{\sigma_{n}^{2}}\exp^{-\frac{v^{2}}{2\sigma_{n}^{2}}}$$

(3) $$f_{s}\left(v\right)=\frac{v}{\sigma_{n}^{2}}\exp^{-\frac{v^{2}+a^{2}}{2\sigma_{n}^{2}}}I_{0}\left(\frac{va}{\sigma_{n}^{2}}\right)$$

We can be seen from Figure 4, selecting the appropriate capture threshold value $V_{t}$ is the key step for signal acquisition to achieve good performance. Too small threshold value can easily cause false alarm, and too high threshold value can easily cause leakage alarm. It is often necessary to set a false alarm rate $P_{fa}$ of signal acquisition and then calculate the corresponding acquisition threshold value according to the false alarm rate. Moreover, signal acquisition sensitivity is also related to false alarm rate and leakage rate $P_{md}$. we assume that the acquisition probability $P_{d}=1-P_{md}=0.9$ and $P_{fa}=10^{-3}$ can be able to capture normally and then the probability density formula is given:

(4) $$P_{fa}=\int_{V_{t}}^{\infty }f_{n}\left(v\right)dv=\int_{V_{t}}^{\infty }\frac{v}{\sigma_{n}^{2}}e^{-\frac{v^{2}}{2\sigma_{n}^{2}}}dv=e^{-\frac{{V_{t}}^{2}}{2\sigma_{n}^{2}}}$$

(5) $$P_{d}=\int_{V_{t}}^{\infty }f_{s}\left(v\right)dv=Q_{1}\left(\sqrt{\rho_{c}},\frac{V_{t}}{\sigma_{n}}\right)$$

where $\rho_{c}=2\frac{C}{N_{0}}T_{c}$ and $Q_{1}\left(\cdot \right)$ is Marcum *Q*-Function. We can get the acquisition threshold can be expressed as:

$$V_{t}=\sigma_{n}\sqrt{-2lnP_{fa}}$$

Simultaneous Equations (4) and (5) with acquisition probability, can be obtained:

$$\rho_{c}=23.65$$

We assume that $T_{c}$ is 1 ms. According to the above equation, we can get the carrier to noise ratio $C/N_{0}$ can be expressed as:

$$C/N_{0}=40.72\;\mathrm{dB}\cdot \mathrm{Hz}$$

At 298 K ambient temperature, the power density of background thermal noise in the atmosphere is −174 dBm/Hz at 1 atmosphere standard, so the acquisition sensitivity is:

$$S_{acq}=C/N_{0}+\left(-174\right)=-133.28\;\mathrm{dBm}$$

In addition, the signal-noise ratio can be denoted as:

$$SNR_{acq}=C/N_{0}\cdot T_{c}=10.72\;\mathrm{dBm}$$

From the above derivation, we can get the conclusion that: (1) the longer coherent integration time can improve the SNR; (2) under the condition of the same false alarm rate, namely the acquisition threshold $V_{t}$ unchanged, the higher signal-to-noise ratio, the higher the probability of detection.

#### 2.3.3. Search Algorithm

**Figure 5:**
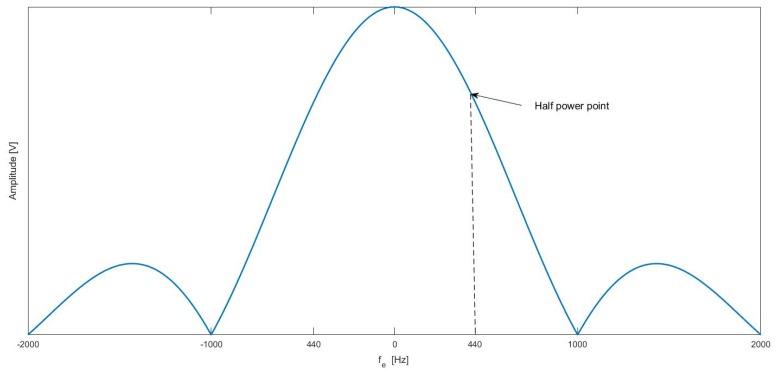
Incoherent integral losses due to frequency differences.

If we assume that a linear search for frequency of signal and the coherent integration time is 1 ms, the simulation result is shown in Figure 5. It can be seen that frequency shift is 0 Hz, and the amplitude V of incoherent integration is the function of |sinc| and 3 dB loss corresponding frequency incoherent integration is about 440 Hz. Therefore, if the coherence integral is 1ms, the frequency interval between 2 frequency without interference is at least 440 Hz, otherwise it is impossible to identify each signal at different frequency.

**Figure 6:**
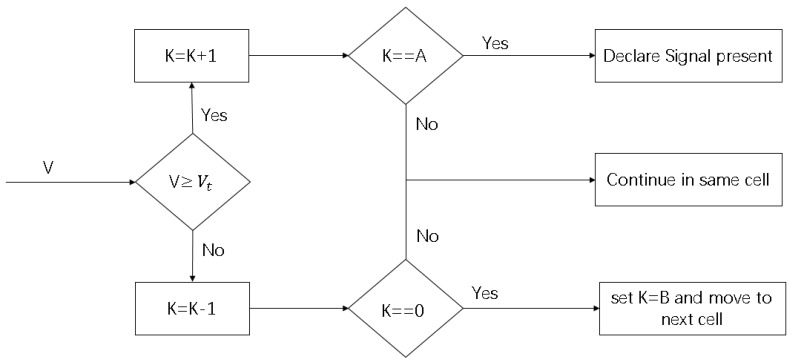
Tong search detector.

Incoherent integral amplitude in the real frequency may be less than other search cell due to noise in range finding signal , so we cannot think that signal acquisition is successful once positive test is obtained and this will also lead to a high false alarm rate. For this reason, we introduce a Tong search detector for PRG method, which is a linear search method in the form of variable search time. As illustrated in Figure 6 [12], the algorithm determines that the search is successful if the detection value of continuous A signal exceeds the threshold value and then it determines that the signal acquisition is successful.

Under the condition that the false alarm rate of a single search is $P_{fa}$ and the detection rate is $P_{d}$ and the overall false alarm rate $P_{F}A$ and the overall detection rate $P_{D}$ can be expressed as:

$$P_{FA}=\frac{{\left(\frac{1-P_{fa}}{P_{fa}}\right)}^{B}-1}{{\left(\frac{1-P_{fa}}{P_{fa}}\right)}^{A+B-1}-1}$$

$$P_{D}=\frac{{\left(\frac{1-P_{d}}{P_{d}}\right)}^{B}-1}{{\left(\frac{1-P_{d}}{P_{d}}\right)}^{A+B-1}-1}$$

### 2.4. Range Signal Tracking

During the range finding signal tracking phase, first the receiver signal channel obtains the carrier frequency estimates from signal acquisition capture phase, and then fine estimates of the carrier frequency and phase is obtained by tracking loop. Finally, the replica carrier is used for phase measurement or transmission to master node.

**Figure 7:**
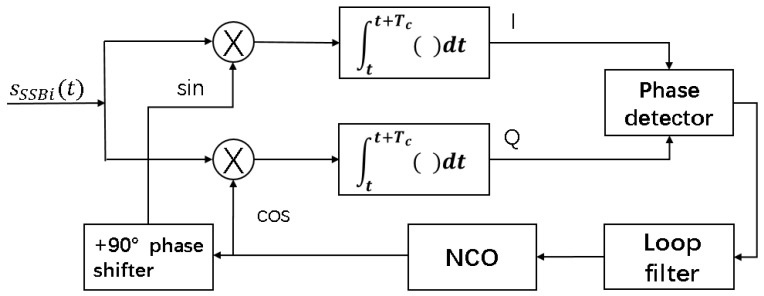
A typical carrier tracking.

The carrier tracking discriminator defines the type of phase-locked loop and frequency-locked loop. The phase-locked loop adopts narrow noise bandwidth and can track the signal closely. The phase precision of the output carrier of phase-locked loop is most accurate, but it is more sensitive to dynamic stress than the frequency-locked loop. The frequency-locked loop adopts the wide noise bandwidth, so it has good dynamic performance and can be more robust when tracking during tolerance of high dynamic stress. However, the signal tracking is slightly less dense due to the higher of loop noise, so the precision of output carrier phase of frequency-locked loop is poor. The main factors influencing the accuracy of ranging in PRG method is carrier loop tracking accuracy to carrier phase. We adopt the phase-locked loop in the carrier tracking loop at the low dynamic scenario. A typical carrier tracking loop is shown in Figure 7.

We can see from Figure 7 that the tracking performance is relate to the phase detector, loop filter and coherent integration time.

#### 2.4.1. Phase Detector

The carrier tracking loop shown in Figure 7 is a Costa loop and the phase-locked loop discriminator commonly used are as follows [12,13]:

1. Two-quadrant arctangent function phase detector:
  $$\phi_{e}=\arctan2\left(\frac{Q}{I}\right)$$
2. Four-quadrant arctangent function phase detector:
  $$\phi_{e}=\arctan2\left(Q,I\right)$$
3. The third method uses the following:
  $$\phi_{e}=\frac{Q}{I}$$
4. Classic Costas analog discriminator:
  $$\phi_{e}=I\cdot Q$$

**Figure 8:**
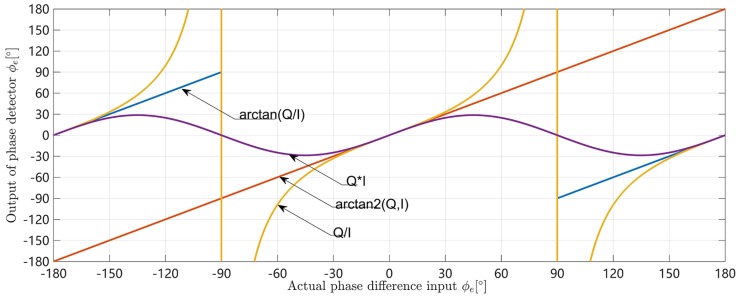
Comparison of PLL discriminators.

Figure 8 compares the input and output relationships of the four phase detectors above without noise.When the actual phase difference is greater than $90^{\circ }$, all the other three phase detectors output phase discrimination results less than 0 except for the four-quadrant arctangent phase detector. This will cause the carrier phase of the tracking loop to adjust to the opposite direction, ultimately causing the loop to unlock the signal.

#### 2.4.2. Loop Filter

To analyze the effect of loop filter on the performance of phase-locked loop, it is necessary to start with the system function of phase-locked loop expressed as:

$$H\left(s\right)=\frac{\theta_{o}\left(s\right)}{\theta_{i}\left(s\right)}=\frac{s}{s+KF(s)}$$

where $\theta_{o}\left(s\right)$ represents the Laplace transform of the instantaneous phase of the output signal of the NCO, $\theta_{i}\left(s\right)$ represents the Laplace transform of the instantaneous phase of the input signal, *K* is loop gain and F(s) is the system function of loop filter.

The selection of F(s) of loop filter is mainly related to the dynamic stress form of users, and the resulting excitation signal can be decomposed into many forms, such as phase step, frequency step and frequency ramp. We assume that this user case does not suffer too much and acceleration effects, so the phase-locked is only affected by phase step and frequency step, so we can choose one order loop filter, which is a second order phase-locked loop, the system function can be expressed by [14]:

(6) $$H\left(s\right)=\frac{2\zeta \omega_{n}s+\omega_{n}^{2}}{s^{2}+2\zeta \omega_{n}s+\omega_{n}^{2}}$$

We can be seen from Equation (6) that the characteristics of the system function is mainly dependent on the characteristic frequency $\omega_{n}$ and the coefficient of damping ζ. From the perspective of frequency domain, the second-order phase-locked loop bandwidth $B_{L}$ can be expressed as:

$$B_{L}=\int_{0}^{\infty }{\left|H\left(f\right)\right|}^{2}df=\frac{\omega_{n}}{2}\left(\zeta +\frac{1}{4\zeta }\right)$$

The selection of loop bandwidth is related to user dynamics and noise performance and the larger the loop bandwidth, the higher the tolerance of user dynamics, but the worse the noise performance. Therefore, the selection of $\omega_{n}$ and ζ needs to balance the noise performance and user dynamic performance. The lower the damping coefficient, the faster the system response to the converges. However, when the damping coefficient is too small, the response of the carrier loop will oscillate violently.

#### 2.4.3. Coherent Integration Time

The coherent integral gain $G_{c}$ is calculated from the change of noise bandwidth before and after the integrator. Although there is no change before and after integral in signal power, noise power spectrum density and carrier to noise ratio, the noise bandwidth $B_{L}$ has changed by the integrator from $B_{pd}$ to $1/T_{c}$. Since the integral gain value is defined as the multiple of the signal-to-noise ratio increase, the integral gain value is equal to the reduction factor of the noise bandwidth and can be denote as:

$$G_{c}=10log\left(\frac{B_{pd}}{1/T_{c}}\right)=10log\left(B_{pd}T_{c}\right)$$

Because there is quantization loss in the process of digital signal processing, which may bring loss to the coherence integral, the actual noise-to-noise ratio will be reduced, generally no more than 2 dB. Next we will analyze the loss of the coherent integral due to the frequency error.

**Figure 9:**
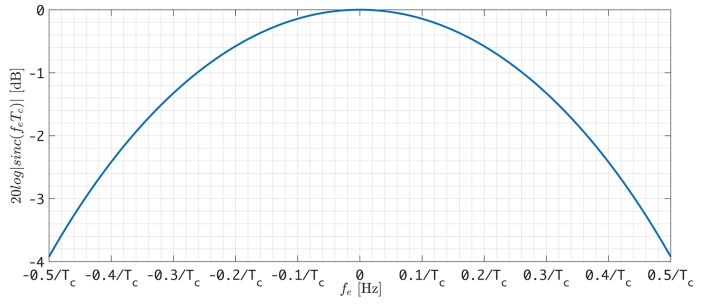
The relationship between coherent integral loss and frequency error.

The coherence integral in the Figure 7 can be denoted as:

$$r_{p}\left(n\right)=I_{p}\left(n\right)+jQ_{p}\left(n\right)=a\cdot \mathrm{sinc}\left(f_{e}T_{c}\right)e^{j\left[2\pi f_{e}\left(t_{1}+\frac{T_{c}}{2}\right)+\theta_{e}\right]}$$

where *a* is the amplitude of IF signal, $f_{e}$ represents frequency error of carrier loop, $T_{c}$ is the coherent integration time, $\theta_{e}$ is the initial phase error and $t_{1}$ is the moment for integration to start. The coherent integral loss caused by frequency error is shown in Figure 9, and we can see from Figure 9 that the 3 dB coherent integral loss is $0.44/T_{c}$, which is also the maximum frequency error $f_{e}$ allowed by the tracking loop.

### 2.5. Coarse Range Finding Method

**Figure 10:**
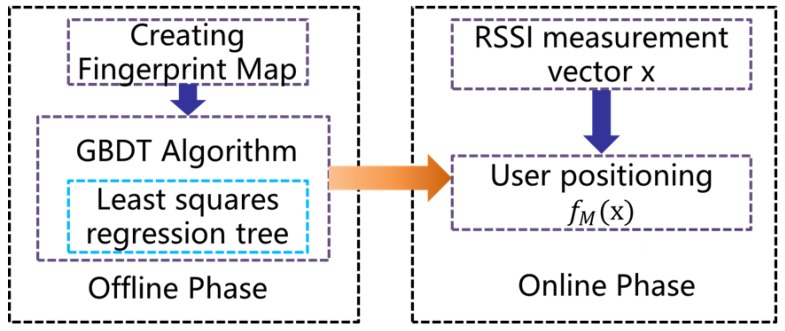
Wi-Fi fingerprint positioning algorithm based on GBDT.

Through the above analysis, we can get the need to provide better than 5λ/12 wavelength ranging precision of coarse range finding method can eliminate the carrier phase integer ambiguity, and then gets the fractional part of carrier phase measurement by the carrier loop measuring phase difference between master and slave node, to can achieve high precision range.We assume that the second-order carrier wavelength of the range finding signal is 30 m, and then the coarse range finding method with accuracy of 12.5 m is required in PRG method. Existing media transmissions such as Wi-Fi, BLE are commonly used for indoor positioning, and can provide the range finding between two nodes. The accuracy of range finding using BLE is less than 1.5 m [6] and Wi-Fi is less than 2.9 m [15]. Range finding accuracy using Wi-Fi and BLE meets the requirements of the above coarse range finding. Wi-Fi signals are common in modern buildings, so there need not deploy any equipment. We can through the fingerprint positioning algorithm based on Wi-Fi get the coarse position of the current node, and then calculate the Euclidean distance of coordinates points of two nodes could satisfy the requirement of the PRG algorithm. We adopted Wi-Fi fingerprint positioning based on Gradient Boost Decision Tree(GBDT), and the algorithm flow is shown in Figure 10.

The proposed fingerprint algorithm consists of two phase: an offline phase and an online phase. During the offline phase, RSS readings and its orientation that the mobile device are facing are collected on grid of reference points(RP), and then these are stored in fingerprint database, GBDT algorithm was used to train the fingerprint database, and the relationship model between signal strength vector x and position coordinate point p was obtained, $f_{M}\left(x\right)$. The online phase consists of the mobile devices measuring RSS, and positioning estimation by model $f_{M}\left(x\right)$.

## 3. Simulation and Analysis

### 3.1. Range Finding Signal Acquisition

**Figure 11:**
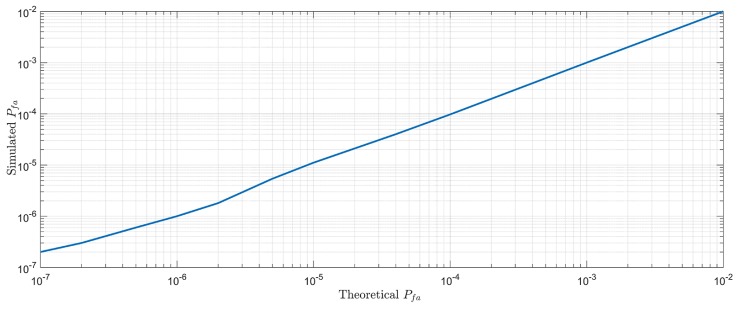
The false alarm rate simulation.

We assume that the signal on the branch of I and Q is subjected to non-correlated gaussian additive noise with a mean of 0 and a variance of $\sigma_{n}$, and we simulated the false alarm rate $P_{fa}$ by monte carlo simulation method. As shown in Figure 11 below, 10,000,000 random points were selected. It can be seen that the theoretical value is very similar to the calculated value.

### 3.2. Distance Measurement Performance of PRG Method

Because PRG method adopts carrier phase to realize distance measurement, its precision is completely dependent on the tracking precision of carrier loop. The tracking accuracy of carrier loop mainly depends on thermal noise, oscillator frequency oscillation error, Allan mean square error and dynamic stress error. In this paper, the simulation experimental parameters are set follows as Table 1, and then the carrier loop tracking error analysis is as follows [10,16].

1. The mean variance of thermal noise error can be expressed as:
  $$\sigma_{tPLL}=\frac{\lambda }{2\pi }\sqrt{\frac{B_{n}}{C/N_{0}}\left(1+\frac{1}{2T_{c}C/N_{0}}\right)}=0.0951\;m$$
2. The mean square error of Allan can be expressed as:
  $$\sigma_{A}=cT_{c}\sigma_{A}\left(\tau \right)=3\times 10^{-3}\;m$$
3. Phase jitter caused by the mechanical vibration of user movement and the receivers of the frequency of oscillation is $2^{\circ }$ , and then
  $$\sigma_{V}=\frac{\lambda }{180}=0.167\;m$$

Then, the 1σ distance measurement error caused by the tracking of carrier loop can be expressed as:

$$\sigma_{PLL}=\sqrt{\sigma_{tPLL}^{2}+\sigma_{A}^{2}+\sigma_{V}^{2}}=0.1992\;m$$

**Figure 12:**
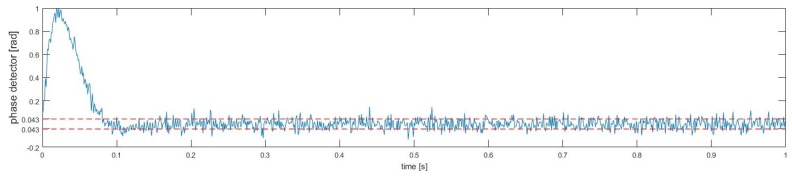
Output of phase detector.

Under the static condition of the user, the parameter setting is the shown in Table 1, and the we use Matlab software to simulate the tracking performance of carrier loop. We can be seen from Figure 12 that after 100 ms, the standard deviation of the tracking phase error is 0.043 rad, and the corresponding tracking error is 0.2055 m. The simulation result is very close to the theoretical analysis above. In addition, the corresponding 1σ range finding error is 0.411 m in the PRG method.

#### Coarse Ranging Finding Performance

In the off-line phase, the fingerprint map is constructed through signal collector, which collects RSSI of each AP through RAK476 module [17]. The positioning area was selected at the room 1015, National University Science Park, Beijing University of Posts and Telecommunications, the total dimension of the room was 5.6 × 7.2 m. In addition, there are multiple deployed wireless routers around the room, and we collected RSSI of 13 reference points in the room. In the off-line training phase, RSSI of 6 AP at 13 reference points was collected within 2 min as fingerprint database, and the grid size was 1.7 m.

**Figure 13:**
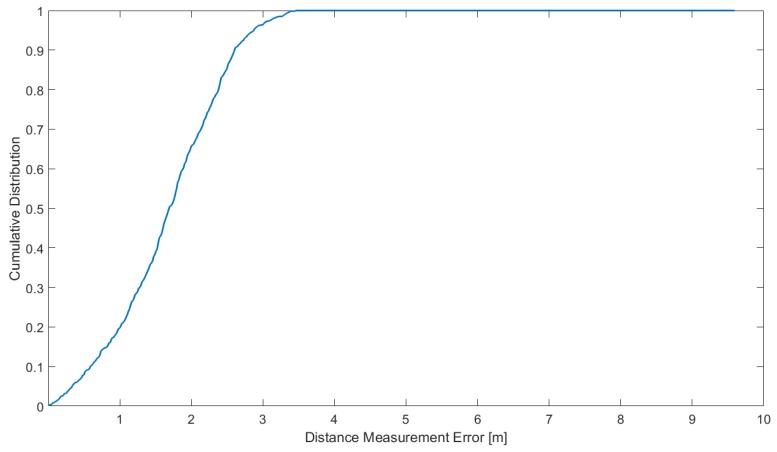
CDF of coarse range finding error.

The cumulative distribution function(CDF) of coarse range finding error is shown in Figure 13, and we can get the range finding accuracy of GBDT fingerprint algorithm with the probability of 67% at 2.05 m and can meet the accuracy of coarse range finding requirements in PRG method.

## 4. Discussion

We suggest that the PRG method can achieve high precision range finding by eliminating the carrier integer ambiguity using coarse range finding method and it provides a basis for high precision positioning. Applying carrier phase measurement to study distance measurement using phase regeneration, we found that the method is both stability and accuracy, as demonstrated in the phase-locked loop results. Distance measurement accuracy was examined by comparing the PRG method and the coarse ranging finding method based on RSS. Our results in the range finding accuracy show a high precision compared to coarse range-based RSS finding method.

The carrier phase measurement technology in PRG method proposed in this paper reference the GPS of measurement technology, and a general standard require the mean square error of carrier phase measurement is less than half wavelength carrier for using pseudorange to round up the carrier phase integer ambiguity algorithm [18], and this requirement is consistent with accuracy of coarse range finding in this paper. Apart from the influence of carrier loop parameter settings on the distance measurement accuracy, the carrier frequency has an influence on accuracy of distance measurement as well. In addition, it is helpful to improve the robustness of PRG method by detecting sudden changes in integer values to cope with large coarse range finding error in practical applications. The higher the carrier frequency, the smaller the spatial distance metric, and then the higher the range accuracy. However, the requirement of precision of coarse range finding also needs to be improved.

The positioning method, based on distance measurement, consists of distance measurement and geometric calculation. Distance measurement can be divided based on the time synchronization and non-synchronization measurement. It is difficult to require high precision time synchronization for positioning in some scenarios, such as IoT. The PRG method proposed in this paper is combined with RTD and carrier phase measurement, so it has the advantages of not requiring high precision time synchronization and high precision distance measurement.

There are two issues that need to be studied in the future. One is the the system design and simulation verification of using Code Division Multiple Access (CDMA) signal as the range finding signal m(t) in multi-master, multi-slave case. The main reason for using CDMA signals is to identify different masters and slaves through different spreading codes. The other is the effect of indoor multipath on the accuracy of range finding by PRG method, we intend to use CDMA signal as range finding signal in the future research, and reduce the influence of indoor multipath through RAKE diversity reception. We plan to use the software radio platform and the existing transmission media (e.g., Wi-Fi, BLE) to achieve ranging by the PRG method, and to research the positioning algorithm in the case of multiple machines. The software radio platform is used to transmit and receive range finding signal and obtain the difference carrier phase between the transmitted and the received replication carrier. Existing transmission media is mainly used for coarse range finding by conventional range finding method.

## Figures and Tables

**Table 1:** Parameter settings of distance measurement performance of PRG method.

Parameter	Value
$B_{n}$	25 Hz
$T_{c}$	1 ms
λ	30 m
$C/N_{0}$	48 dB· Hz

## Abbreviations

The following abbreviations are used in this manuscript:

PRG	Phase Regeneration
IoT	Internet of things
GBDT	Gradient Boost Decision Tree
RSS	Received signal strength
RSSI	Received signal strength indicator
PLL	Phase-locked loop
FLL	Frequency-locked loop
CDF	Cumulative distribution function
GPS	Global Positioning System
RTD	Round-trip delay time
